# Measuring and Training Speech-Language Pathologists' Orofacial Cueing: A Pilot Demonstration

**DOI:** 10.1155/2018/4323046

**Published:** 2018-12-31

**Authors:** Aravind Kumar Namasivayam, Rohan Bali, Roslyn Ward, Krystal Danielle Tieu, Tina Yan, Deborah Hayden, Pascal van Lieshout

**Affiliations:** ^1^Oral Dynamics Lab, Department of Speech-Language Pathology, University of Toronto, Toronto, Ontario, Canada; ^2^Toronto Rehabilitation Institute, Toronto, Canada; ^3^Department of Paediatric Rehabilitation, Princess Margaret Hospital for Children, WA, Australia; ^4^Faculty of Medicine, School of Paediatrics and Child Health, Dentistry and Health Sciences, The University of Western Australia, Perth, WA, Australia; ^5^The PROMPT Institute, Santa Fe, New Mexico, USA; ^6^Rehabilitation Sciences Institute, University of Toronto, Toronto, Ontario, Canada

## Abstract

Tactile-kinesthetic-proprioceptive (TKP) input used to facilitate speech motor control is considered an active ingredient within speech motor interventions. Objective metrics identifying skill level differences across speech-language pathologists (S-LP) providing TKP cues are crucial for monitoring treatment delivery fidelity. The study examined three kinematic measures indicating accuracy and consistency of TKP inputs by 3 S-LPs with varying experience levels (S-LP 1: novice; S-LP 2 and S-LP 3: advanced). Confidence interval measures were used to compare the accuracy of jaw movement amplitudes of the vowel /a/ made by a model participant versus S-LPs giving the TKP input. Generalised Orthogonal Procrustes Analysis (GPA) and cyclic Spatial Temporal Index (cSTI) were used to determine movement consistency. Results revealed passive jaw excursions induced by S-LP 2 and 3 to be not statistically significant from the model participant's active jaw movements. cSTI values decreased with advanced level of experience (19.28, 12.14, and 9.33 for S-LP 1, S-LP 2, and S-LP 3, respectively). GPA analyses revealed a similar pattern for S-LPs with more experience demonstrating lower mean RMS values (0.22, 0.03, and 0.11 for S-LP 1, S-LP 2, and S-LP 3, respectively). Findings suggest kinematic measures adapted from the motor control literature can be applied to assess S-LP skill differences in providing TKP cues.

## 1. Introduction

The use of tactile-kinesthetic-proprioceptive (TKP) inputs has an established presence in remediating speech sound disorders (SSDs) within many contemporary speech motor interventions (e.g., [1, 2]. Typically, therapists provide TKP cues to a client's orofacial structures via direct manipulation of mandibular movement and by targeting certain orofacial and lingual muscles [3]. These techniques emphasize place, voice, and manner of speech production, as well as slowing down speech rate to enhance proprioceptive feedback and highlight movement transitions [3].

The effectiveness of TKP cues in improving accuracy of words and phrases, facilitating movement generalization, and establishing, refining, and integrating normalized speech movement patterns within several articulatory subsystems (e.g., mandibular, labial-facial, and lingual) has been demonstrated in a number of behavioral studies (e.g., [1, 4, 5]. TKP inputs are said to take advantage of the plasticity of the sensorimotor cortex [6, 7], by broadening sensory-motor map representations, facilitating motor performance, and expanding client behavioral repertoires [8, 9].

Given the importance of TKP inputs in sensory-motor mapping and speech movement stability (see [10] for a discussion on the role of variability in speech motor control), it is crucial for S-LP's providing TKP inputs to be accurate and consistent. A speech-language pathologist (S-LP) must learn new and specific hand orientations, trajectories, finger placements, timings, and pressure inputs to effectively provide relevant TKP cues. Research in other disciplines has recommended the active measurement of provider skill acquisition [11–13]; however, there is scarce literature in the area of communication disorders on the assessment of training effectiveness, standardization of clinical procedures, and reporting of treatment fidelity (e.g., [14, 15]).

However, some fidelity assessment procedures have been described and utilized by researchers in communication disorders [13, 16–18], and specific assessment procedures evaluating new skill acquisition in S-LPs regarding accurate and consistent implementation of TKP cues do not exist [16]. How might one objectively assess an S-LP's ability to learn and provide accurate and consistent TKP cues with training? From a general motor control and motor skill acquisition perspective [19], we can possibly identify objective measures to track differences and changes in skill levels in S-LPs.

There is consensus among a number of theoretical perspectives that learning complex skills is a process occurring over several time scales [20] and involves three stages: (a) assembling a coordination pattern, (b) gaining control of a coordinative structure, and (c) skilled optimization of control [21]. In general, motor skill acquisition is evident through practice-driven improvements in movement accuracy and consistency (i.e., decrease in movement variability) when context and task conditions remain constant [19–21]). Thus, it would be possible to estimate differences in fine motor skill levels in SLP's providing TKP cues by measuring the variability of their finger-hand movements and end-product changes (i.e., movement changes in target structures such as the lips and jaw in a client/participant's face). Importantly, an S-LP must master a specific motor skill (i.e., providing the TKP inputs) and accurately observe the appropriateness of each client's speech motor responses (i.e., perceptual skill) in order to plan appropriate interventions [1]. By utilizing a task that requires making judgements of jaw movement ranges in a client, it is possible to investigate this visual perceptual acuity in S-LPs. Thus, the exploration and identification of dependent variables (related to perceptual sensitivity and movement execution) to serve as objective metrics of differences in skill levels in S-LPs providing orofacial TKP cues is important, not only for monitoring fidelity in treatment delivery and benchmarking of service quality but also for assessing the readiness of trainee S-LPs for unsupervised practice [15].

The current study sought to evaluate whether three objective kinematic measures relating to the accuracy and consistency of S-LP hand movements and the way these movements are able to match resulting passive orofacial movements to active (natural) movement ranges can be used to distinguish experience levels of S-LPs providing those TKP cues. For the purpose of this pilot study, a model participant without speech problems was deemed to provide a more reliable context for testing the usefulness in assessing clinicians' skills. We hypothesize that, with more specialized training and years of experience with speech motor disorders, a S-LP should (a) demonstrate increased accuracy when predicting and cuing client jaw ranges for a given vowel (relative to the 95% confidence intervals (CIs) of the client's mean range of active jaw movements), (b) demonstrate increased consistency in finger-hand movements when providing TKP cues as determined by Generalised Orthogonal Procrustes Analysis (GPA), and (c) demonstrate increased consistency in induced (passive) orofacial movements using a cyclic Spatial Temporal Index (cSTI) measure. These measures will be explained in more detail in the method section.

## 2. Method

### 2.1. Data Acquisition

A 23-year-old female with no self-reported speech, language, hearing, or neurological difficulties served as the model participant-client in this study. Three female S-LP participants, with different levels of training and experience (S-LP 1: novice; S-LP 2 and S-LP 3 advanced), provided TKP cues to the model participant's orofacial structures. S-LP 1 had recently graduated from an accredited S-LP program with eight months experience providing S-LP services to children with speech sound disorders. S-LP 2 and S-LP 3 had more than 15 years of experience and advanced training with speech motor interventions with both children and adults. The study was approved by the University of Toronto's Health Sciences Research Ethics Board, and all participants provided a written informed consent prior to participation.

All kinematic and time-aligned acoustic data were collected with the Electromagnetic Articulograph 501 system (EMA AG501) [22]. The EMA AG501 consists of a three-armed structure with 9 transmitter coils located above the participant's head used to generate alternating electromagnetic fields, each at their own oscillating frequency. When sensor coils are introduced into the field, a weak current is induced in the sensor coils, with the signal strength proportional to the distance of the sensor from the transmitters and its orientation. This allows tracking of the spatial positions of the sensor coils which are calculated by using the *CalPos* program available from the manufacturer [22]. The median errors for the AG501 systems are under 0.5 mm [23, 24]. All movement data were recorded at 250 Hz, whereas acoustic signals were sampled at 48 kHz. Data from the EMA system were low-pass filtered to 10 Hz, removing noise from the movement signals [25]. Sample-by-sample head movement correction was carried out by rotating and shifting the coordinate system based on the reference sensors located on the participant's left and right mastoid and nose bridge (e.g., [23]). The movement data were processed with the “EGUANA” Matlab toolbox [26] to extract dependent variables.

Sensors were attached onto the model participant's face at the following anatomical locations: mandible midline, nose bridge, behind the left and right ear mastoid, and two sensors 1.5 cm symmetrically on either side of the philtrum of the upper lip. Furthermore, six sensors were placed on the hand providing the TKP inputs in the following manner: one each on the thumb, index finger, and middle finger (placed on the nail plate) and three reference sensors on the dorsal part of the hand.

Speech stimuli were vowels /a, i, u/ produced in isolation and in a sequence combined with the consonant /t/ as in /ta, ti, ta, tu/ in succession. Movement data were recorded under two conditions: (a) *active condition*: the model participant repeated the stimuli (vowels in isolation and in sequence) approximately 10 times at a self-paced rate and (b) *passive condition*: the model participant's movements were generated solely by the action of the S-LP's hand movements; e.g., moving the jaw down (e.g., for vowel /a/) or rounding/retracting the participant's lips (more detail provided below). The order for participant-S-LP dyads was S-LP 3, S-LP 1, and S-LP 2.

### 2.2. Measures

Three kinematic measures were used to quantify accuracy and consistency of TKP cues provided by an S-LP. All of these measures have been modified and adapted from measures previously reported in the speech and limb motor control studies [25, 27].

#### 2.2.1. Measuring Accuracy of Induced Jaw Position

**Figure 1:**
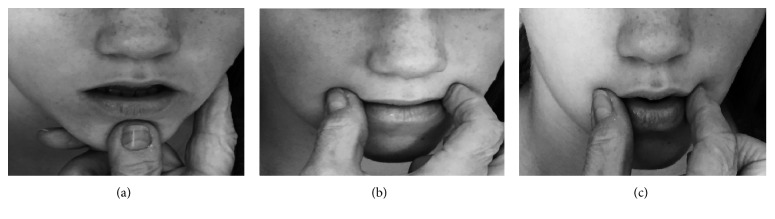
S-LP finger placement and orientation for TKP inputs related to vowel /a/ (a), vowel /i/ (b), and vowel /u/ (c).

Measuring accuracy of jaw movements has been deemed of crucial importance in enhancing the critical role of jaw control in speech production [28, 29]. Deviances in jaw stability and control have been reported in children with SSDs [30], for example, a tendency to lower vowel jaw movement range (e.g., /Ɛ/ -> /æ/) and/or produce diphthongs as monophthongs [31]. Clinically, in approaches such as PROMPTs for Restructuring Oral Muscular Phonetic Targets (PROMPT) [3], jaw control issues are addressed by the therapist manipulating the client's jaw to reach the appropriate jaw range for a target vowel, followed by stabilization of the jaw prior to targeting lip-tongue movements in therapy [3]. Critical to this process is the therapist's ability to accurately estimate the optimal jaw location for each target vowel for each client's own orofacial dimensions, muscle tension, and jaw range. In the current study, we designed an accuracy measure to capture this clinical skill by comparing the 95% CIs for jaw movement ranges derived from the model participant's active productions for a particular vowel (/a/) with S-LP induced passive jaw movements. For this, the S-LP's thumb was placed on the participant's chin, while the bent middle finger was placed under the chin for support (Figure 1). The index finger was placed along the jaw line. Jaw movement range for high vowels (/i/ and /u/) was not analyzed in the current study as they were produced with very little measurable jaw movement. Means and SD were calculated from the 10 repetitions each of passively and actively generated jaw movements by the participant for vowel /a/ (Table 1). Accuracy was estimated by plotting the passively generated jaw movements for vowel /a/ over the 95% confidence intervals (CIs) derived from the model's active jaw movements. Mandibular measurements were based on the position of the mandibular sensor coil.

#### 2.2.2. Measuring Consistency of Hand and Induced Upper Lip Movements

Two measures were utilized to quantify the consistency of TKP inputs provided by an S-LP: the Generalised Orthogonal Procrustes Analysis (GPA) and the cyclic Spatial Temporal Index (cSTI). For vowel /i/, the thumb and index fingers were placed above the upper lip at the intersection between levator anguli oris and the skin locations covering the location of zygomatic major muscles with slight pressure applied backwards towards the model participant [32] (Figure 1). This results in lip retraction. For vowel /u/, the thumb and index fingers were placed at the lip corners at the intersection between zygomatic major and orbicularis oris muscles, with the direction of movement outwards in lip protrusion (Figure 1). Since vowel /a/ has no specified target for either rounding or retraction, this vowel was not considered in this analysis.

#### 2.2.3. Generalised Orthogonal Procrustes Analysis (GPA) for S-LP Hand Movements

Generalised Orthogonal Procrustes Analysis [33, 34] has been used in motor control studies, including for the measurement of consistency of arm movements in reaching tasks [27, 34]. We utilized GPA to assess consistency in the shape of the S-LP's finger space movement paths during delivery of TKP cues. Movement of the S-LP's fingers on the model participant's face is influenced by a number of external and internal factors. The S-LP may adjust their hand orientation (tilt/rotate) in three dimensions to compensate for the model participant's head position or location. Additionally, subsequent repetitions of the S-LP's finger movements may differ in their duration and amplitude. Though the general shape may be the same, the paths of the S-LP's hand may vary slightly in location, orientation, and scale, referred to as extrinsic variability [34]. Shape is defined as the geometrical information that remains consistent when location, scale, and rotational effects (extrinsic variability) are filtered from an object [35]. What remains reflects intrinsic variability of the finger-hand paths in three-dimensional (3D) space. GPA rotates and transforms the movements measured from each repetition of a stimulus, superimposing them on one another by least-squares fitting. Each repetition of the 3D finger-hand movements (henceforth referred to as shape) is aligned to a common mean or consensus representation [34]. It has been suggested that this mean shape following the GPA represents an underlying invariant shape or template that the motor system is attempting to execute, and the intrinsic variability around that mean shape indicates precision (or alternatively, difficulty) in executing that template [34].

The entire movement trajectory data from the S-LP's thumb (relative to the reference sensor on the participant's nose bridge) over successive trials of stimuli “ta-ti-ta-tu” was used for the GPA analysis. To extract movement data of the S-LP's thumb relative to the reference sensor, the latter was subtracted from the trajectory of the S-LP's thumb in 3 dimensions (X = front/back, Y = left/right, and Z = up/down). Before applying GPA, each movement trajectory was time normalized to 1000 points using FFT (FFT is an algorithm that completes the discrete Fourier transform of a sample of points. During FFT interpolation, a sequence of values across time is resolved into the frequency domain using the FFT algorithm. These frequencies are then converted back into the time domain using inverse Fourier transform, and however, the sampling frequency is kept different from that initially used. In this manner, we are able to change the sampling frequency (hence number of points) in a time-domain signal, while preserving the underlying frequency values of the signal itself) interpolation separately for each of the three dimensions, across 10 repetitions of /ta-ti-ta-tu/. A centroid or mean value was calculated for each movement trajectory and for each dimension (X, Y, and Z). The movement trajectories were then linearly shifted such that the centroids are aligned with the origin (noise bridge sensor coil). The thumb movement data for each of the 10 repetitions of /ta-ti-ta-tu/ was then represented as a 1000∗3 matrix, with each of the rows representing 3D positions at a particular time and each column representing the position value along one of the three Cartesian coordinates across time. For convenience in describing the GPA method, we will follow the convention of referring to each of the 10 1000∗3 matrices as Si (where i represents the ith repetition). During the GPA process, we attempt to align repeated 3D movements. In our case, we have 10 repetitions. Each repetition contains a 1000 points of 3-dimensional data (hence a 1000∗3 matrix). Si is a convention to represent one such matrix. S1 would represent the first matrix representing the first repetition, S3 would represent the third matrix representing the third repetition, and so on.

For the GPA analysis, one movement trajectory of the 10 repetitions of /ta-ti-ta-tu/ was selected as a common consensus trajectory or exemplar (M), and the remaining nine movement trajectories were rotated to align with this exemplar. Matrix rotation is performed by finding an orthogonal matrix Qi for each movement trajectory Si which minimizes the value of ||Si∗Qi-M||. The solution for finding such an orthogonal rotation matrix Qi has been published elsewhere [36]. For the rotated trajectories, a mean trajectory (M; which is used to replace the original exemplar) is then calculated in 3D space. This process of calculating the mean trajectory and rotating/aligning is repeated with a new and updated consensus trajectory M until the difference between the mean trajectories is less than a threshold value across subsequent iterations. The resultant trajectories derived from the repeated rotations of the movement trajectories are referred to as shapes. In order to find the intrinsic variability in these shapes, we calculated the mean RMS (the RMS error is defined as the root mean square error. If Si is the ith repetition and Si(j) is the jth value on this repetition (where *j* goes from 1 to 1000), rms of the ith trajectory (rms(i)) from the mean is defined as square root (((Si(1) − Smean(1))^2^ + (Si(2) − Smean(2))^2^ … (Si(1000) − Smean(1000))^2^)/1000). The mean RMS is therefore defined as (rms(1) + rms(2) … rms(10))/10 for 10 repeated trajectories) residual between each of the shapes and their mean consensus trajectory. The RMS residual for one trajectory is calculated by summing the squared distances of each data point in the trajectory to the corresponding data point in the consensus trajectory, averaging over the number of data points (1000) and calculating the square root. The mean of this RMS residual over all the movement trajectories derived from a S-LP's thumb sensor coil gives us a measure of the intrinsic error in the thumb movement pattern (or shape) of that S-LP. Additionally, to account for differences in S-LP's range of hand motion, we also report displacement-normalized RMS residuals (nRMS).

#### 2.2.4. Cyclic Spatial Temporal Index (cSTI) for Induced Upper Lip Movements

However, GPA assesses consistency in the shape of the S-LP's finger space movement paths, and we need a measure to capture end product consistency of a S-LP's finger-hand movement trajectories (i.e., the resulting changes in the orofacial structures, for example, skin stretch or deformation). In the current study, cyclic STI or cSTI [25] was used based on the more general STI measure developed by Anne Smith and his colleagues [37]. The cSTI reliably measures consistency of speech articulatory movement cycles across repeated productions [10]. The underlying assumption is that highly practiced and consistent movement cycle trajectories normalized in amplitude and time converge upon a single core template. The higher the cSTI value, the greater the deviation from a single template [25]. cSTI was measured based on data derived from the distance between two sensor coils placed on a model participant's upper lip as the S-LP-induced upper lip retraction gestures (e.g., as used during the production of the vowel /i/).

Kinematic consistency of passively induced upper lip movements was indexed using the cSTI, with movement cycles operationally defined as peak-to-peak or valley-to-valley trajectory cycles related to the Euclidean distance between the two sensor coils placed 1.5 cm symmetrically on either side of the model participant's philtrum on the upper lip. For cSTI, cycles were limited to displacement records for /ti/ for which the S-LP was inducing lip retraction gestures in the model participant. Lip gestures corresponding to /tu/ (for lip rounding) were not used in the cSTI analysis due to motion artifacts caused by lip muscle protrusion and bunching (resulting in sensor coil rotation) from the S-LP's fingers pulling the lips closer. The lip retraction displacement records for /ti/ gestures were time (to 1000 points) and amplitude (*z-*score transformed) normalized. From these records, 50 standard deviations were obtained at 2% intervals on the normalized axis and summed to give the cSTI score [25].

Descriptive statistics (Mean and Standard Deviations (S.D.)) for passive jaw excursions (in mm) induced by S-LP 1, 2 and 3 compared to active jaw movements made by the model participant are provided in Table 1. For measuring accuracy of induced jaw position statistical significance was tested using an unpaired *t*-test Bonferroni corrected for multiple comparisons (adjusted *p* value 0.05/3 = 0.01) using an online biostatistics software (http://graphpad.com/quickcalcs/ttest2/).

## 3. Results

### 3.1. Accuracy of Induced Jaw Position

**Figure 2:**
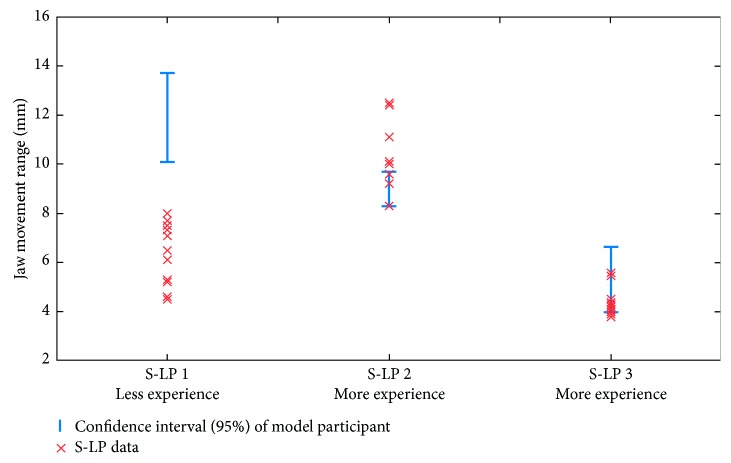
Accuracy data across 3 S-LPs. Confidence Interval (95%) of jaw movement range for actively produced vowel /a/ by the model participant compared to induced movement ranges by the 3 S-LPs. Each cross represents an S-LP attempt, approximately 10 attempts per S-LP.

Jaw accuracy data (Table 1) revealed that S-LPs 2 and 3 were better able to induce passive jaw excursions within the 95% CIs of the model participant's active jaw movements when actually making the intended vowel sound, compared to the less experienced S-LP 1 (Figure 2). Unpaired *t*-tests indicated that, for S-LPs 2 and 3, passive jaw excursions induced were not statistically different from actual active jaw movements made by the model participant (Table 1). Figure 2 indicates amplitude differences in the jaw movement range for actively produced vowel /a/ stimuli varied across the test sessions (as each S-LP was run on a different day). Thus, regardless of the absolute amplitude of the model participant's active jaw movements, only the experienced S-LPs matched the required movement range after viewing these active productions.

### 3.2. Consistency Findings for Hand and Induced Upper Lip Movements

#### 3.2.1. GPA

**Figure 3:**
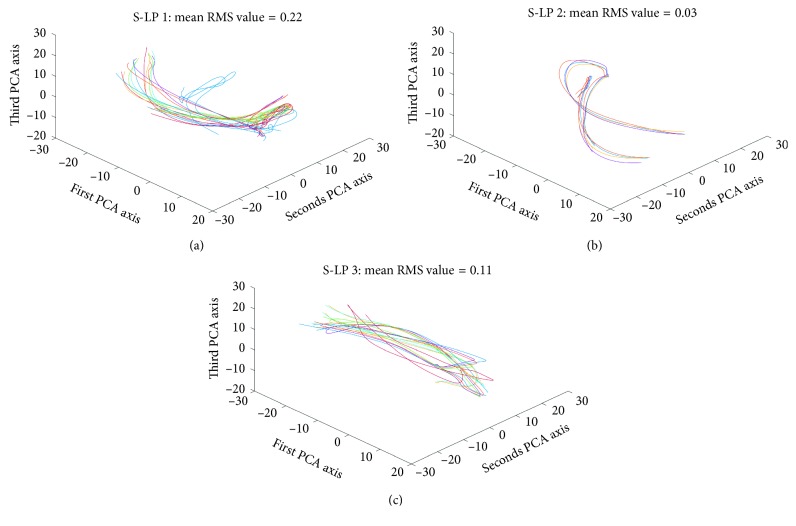
GPA analysis as applied to S-LP's thumb finger movement trajectories over successive trials of stimuli “ta-ti-ta-tu”. Since the movement paths are rotated, the trajectories after GPA do not correspond to the original movements in the Cartesian axis. Hence, by convention, we use the principal axes of the GPA consensus path in the plots. Data from (a) S-LP 1, (b) S-LP 2, and (c) S-LP 3.

In Figure 3, GPA analysis revealed intrinsic variability of the thumb paths of the three S-LPs was different. S-LPs with more experience demonstrated lower mean RMS residual values (S-LP 2 GPA = 0.03 and S-LP 3 GPA = 0.11; Figures 3(b) and 3(c), respectively) relative to the S-LP with the least experience (S-LP 1 GPA = 0.22; Figure 3(a)). A similar pattern emerges for displacement-normalized RMS residuals, with S-LP 2 and S-LP 3 demonstrating lower values (S-LP2 = 0.00012; S-LP3 = 0.0007) than S-LP 1 (0.0012).

#### 3.2.2. cSTI

**Figure 4:**
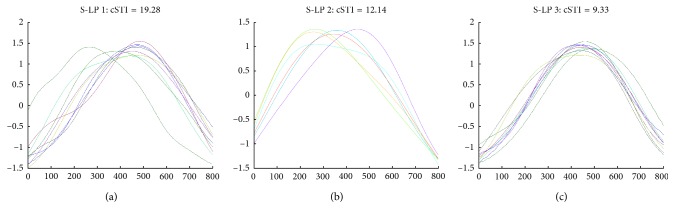
Depicts cSTI values derived from the amplitude- and time-normalized displacement records (for /ti/- lip retraction induced by S-LP) from the two sensor coils placed 1.5 cm symmetrically on either side of the model participant's philtrum on the upper lip (note: 100 points from the onset and offset were removed from analysis due to high frequency artifacts arising from windowing). Data from (a) S-LP 1, (b) S-LP 2, and (c) S-LP 3, respectively.

In Figure 4, cSTI values relating to passively generated (i.e., induced) upper lip-retraction movements indicate that S-LP 1 with the least experience had the highest cSTI values at 19.28 (Figure 4(a)), whereas cSTI values for S-LP 2 (Figure 4(b)) and S-LP 3(Figure 4(c)) were 12.14 and 9.33, respectively.

## 4. Discussion

The purpose of the study was to identify objective measures to distinguish SLP's skill levels in providing TKP cues. We investigated whether three kinematic measures relating to the accuracy and consistency of S-LP hand movements and the resulting model participant's passive orofacial movements would distinguish experience levels of S-LPs providing TKP cues. Overall, we found that, in the task of accurately estimating a client jaw movement range, changes in consistency of an S-LPs own finger-hand movements when providing TKP cues and of the induced (passive) orofacial movements in a model participant varied as a function of the S-LP's training and experience. These findings suggest that kinematic measures adapted from the speech and limb motor control literature can be successfully applied to quantify S-LP skill levels in providing TKP cues. The broader clinical implications of these findings will be discussed next.

### 4.1. Accuracy Findings for Induced Jaw Positions

The act of speaking requires coordination of the speech subsystems (respiration, phonation, and articulation) within time and space. Past literature has shown that the multiple degrees of freedom of movement in speech production are constrained through organized functional synergies, allowing for variability in movement patterns [10, 38]. Hence, an S-LP utilizing TKP cues must achieve mastery of a specific motor skill (i.e., providing the TKP inputs) and the ability to observe each client's motor response. Data in this study show two experienced S-LPs were able to match the model participant's active jaw amplitude changes using TKP cues, whereas the inexperienced S-LP was less able to do so, affirming the need to train towards achieving an appropriate level of skill.

Fundamental to intervention for clients with SSDs are skills requiring attunement to task-specific visual information while observing a client's ability to execute a speech motor movement, assessing where difficulties are occurring, and planning intervention by selecting speech motor targets using TKP inputs [1]. In our study, differences observed between more- and less-experienced S-LPs can be interpreted in the context of the visual search strategy paradigm [39]. Within this paradigm, visually extracting meaningful information requires an experienced knowledge base of the most relevant information areas (for clinician attention) and the least relevant areas (to be ignored) [39]. For example, a S-LP needs to extract information of a client's age and physical anatomy, interpret the accuracy of a client's movement patterns regarding the task at the hand, and apply this knowledge within the context of speech motor control principles. A recent meta-analysis of sports science research reported that experts and novices fixate in different areas of a given visual display [40]. Mann and his colleagues concluded that search strategies are guided by task-specific knowledge structures stored in memory and developed from experience with similar and related situations. Our findings seem in agreement with this assertion.

More specifically, for S-LP training, the S-LP needs to learn how to perform the motor act underlying the TKP cues, that is, learn to move his/her hand/fingers to a specific spatial location (i.e., client's jaw/face), timed with the movement within the intended range [41]. This is consistent with findings from Koedijker and his colleagues [42] that novices need to consciously monitor and control their movements, whereas experts have attained a higher level of automaticity and no longer need to engage in conscious monitoring.

### 4.2. Consistency Findings for Hand and Induced Upper Lip Movements

Contemporary motor control literature considers the consistency with which behaviors are repeatedly executed to be one of the critical elements of skilled motor expertise [43, 44]. Variability is considered a functional exploratory behavior that enables an individual to enhance their motor performance during skill acquisition [43–45]. That is, during the initial stages of skill acquisition, a learner will explore a number of different strategies before selecting the best solution [46]. Reduction in variability (i.e., consistency) for a given movement pattern is considered the identification of a stable solution based on a learner's attunement to key information sources (i.e., task, environment, and individual) and represents the emergence of a specialized skill [46]. Our consistency measures (GPA and cSTI) reflected this pattern as a function of S-LP skill and training level.

#### 4.2.1. GPA for Hand Movements

The GPA is said to reflect the precision or computational difficulty with which the motor system executes the desired movement paths. The higher mean RMS residual values in S-LP 1 (least experienced) may imply a greater difficulty in planning and executing complex thumb movement paths as required for TKP inputs in a therapeutic context. The data from the more experienced S-LPs demonstrated that they executed their finger/hand movements with greater precision, indicated by the lower RMS values. In a study by [47], principle component analysis was used to evaluate right arm movements during bowing in novice and expert cello players. 3D kinematic data showed higher variability in the coordination of the degrees of freedom of movement of novice musicians and statistically significant differences in the use of shoulder versus elbow, wrist, and finger movements. Experts moved in a systematic and temporally coupled manner, whereas novices did not. Thus, experts not only showed better control and less variability but also higher skill acquisition. The data from our study are consistent with the findings of Verrel et al. [47] and with the motor control literature generally [43, 44].

#### 4.2.2. cSTI for Induced Upper Lip Movements

From a clinical standpoint, consistency of induced movements in target structures such as the lips and jaw (i.e., end product of finger-hand cueing movement trajectories) is as important as consistency of S-LP hand movements. Experimental data suggests that somatosensory signals arising from cutaneous afferents in the facial skin (in the absence of muscle receptors in the perioral structures) play a crucial role in speech motor learning and adaptation [48, 49]. For example, externally applied skin stretch or deformation of perioral structures especially lateral to the oral angle (as in the current study (Figure 1)) has been shown to affect somatosensory signals from cutaneous afferents. Such signals are critical for the detection and control of lip and jaw articulatory motion and perceptual processing of speech sounds [49]. These cutaneous mechanoreceptors in the facial skin lateral to the oral angle are narrowly tuned, and hence, inconsistent and/or inaccurate TKP cueing may impact kinesthetic information relevant for rapid sensorimotor processing of speech [48]. In the current study, cSTI data revealed that end-product consistency varied as a function of S-LP experience; the S-LP with the most experience induced the least variable passive movements, whereas the S-LP with the least experience created more variable passive movements. Our results show preliminary but positive support for the use of kinematic measures to distinguish motor skill levels of S-LPs providing TKP cues relevant for attunement to active movements, providing consistent hand movements, and the consistency of induced articulatory movements.

### 4.3. Implications for Training and Treatment Fidelity

The importance of establishing treatment fidelity to determine treatment efficacy has been clearly established [17]. In speech and language literature, Hayden and his colleagues [16] have reported that PROMPT-trained clinicians are required to achieve a fidelity rating of greater than 80% for the intervention to be considered in accordance with the planned intervention prototype. However, there is little research on how trainees might acquire these necessary skills. Learning a complex motor skill such as the accurate presentation of TKP inputs will be characterized by changes over time [50] and may take years to achieve mastery or treatment fidelity. Experience levels of S-LPs in this study ranged from 8 months to 35 years, with past literature frequently reporting expert performance (across a range of tasks) to require approximately 10 years [51, 52].

Verrel et al. [47] postulated that motor control demonstrated by expert cellists in their study may have been associated with the explicit teaching of the variables evaluated within their experiment. Thus, learning complex fine motor skills may quicken when learners are provided with specific and necessary feedback along with adequate training opportunity. There is a clear need for further research to identify the specific aspects of TKP cueing for improving client speech productions. This will not only include better understanding of the learner's expertise but also the functional skills to be trained and primary learning constraints [46]. These preliminary findings are a first step for further explorations of the feasibility of these measures for evaluating S-LP functional skill levels in training settings.

### 4.4. Study Limitations

The study has several major limitations. External generalizability of these results is severely limited by the small sample of participants. A demonstration of these measures and outcomes with a larger group of participants is imperative. Furthermore, using a healthy model's actual facial movements to judge appropriate TPK cueing, although appropriate for the context of this pilot study, does not allow for generalization to clients with impaired speech production skills. In the field of speech-language, pathology assessment of functional speech outcomes is critical. In therapy where these cues are typically used, such measures are indeed critical for assessing progress in a client's speech production abilities. However, this information was not gathered in the present study as the focus was specifically on the accuracy and consistency measures obtained from the S-LP's hand movements and S-LP-induced passive jaw and lip movements on a model participant's face. This individual was not actually speaking during measurements of these variables (other than to establish the jaw movement range), again because our focus for the present study was not on the acoustic outcomes but on the delivery of tactile cues. This is why, for the purpose of this pilot study, we used an adult model participant without speech, language, hearing, or neurological issues so it would be possible to access the specific features of tactile cue delivery without possible confounding issues presented by a motor speech disorder. This presented a more reliable context for testing the feasibility of assessing clinicians' skills. In a healthy adult participant with perfectly intact and “normal” functioning sensory-motor systems, we do not expect any significant changes in speech acoustics as a function of accuracy and consistency of TKP cues, especially with a limited number of trials (10 repetitions of target items). However, in the context of a developing sensory-motor system in a child or an impaired speech system in either adults or children (e.g., subsequent to brain injury), the auditory-to-speech motor mapping required for the achieving accurate speech output may be more susceptible to inaccurate and inconsistent TKP input by an S-LP. The findings from the current study suggest that kinematic measures adapted from the motor control literature can indeed be applied to assess S-LP skill differences in providing TKP cues. Utilizing these objective measures to capture S-LP skill levels, future studies must be conducted on populations with speech disorders to establish the impact of S-LP skill levels on functional speech outcomes.

Lastly, the cross-sectional design used provides no indication of the longitudinal development of motor skill in clinicians and limits causal inference. Future studies should track S-LPs' motor skill levels as a function of training in a longitudinal study using a population with specific speech disorders and possibly within a therapeutic context. This may one day allow us to track S-LPs' motor skill levels objectively and set fidelity standards for providing specialized speech treatments.

## 5. Conclusions

Overall, we found that accuracy in estimating and subsequently implementing a client's jaw movement range, consistency in finger-hand movements when providing TKP cues, and the consistency of the resulting-induced (passive) orofacial movements in a model participant varied as a function of the S-LP's training and experience. These findings suggest that kinematic measures adapted from the speech and limb motor control literature can be successfully applied to quantify S-LP skill levels in providing TKP cues. These preliminary findings are a first step for further explorations of the feasibility of these measures in evaluating S-LP functional skill levels during training and for determining if a clinician is ready to treat patients without supervision.

## Figures and Tables

**Table 1:** Descriptive statistics mean (standard deviation) and number of attempts (*N*) for passive jaw excursions (mm) induced by S-LP 1, 2, and 3 compared to active jaw movement ranges made by the model participant for the same vowel (/a/) at three different sessions.

S-LP	Participant	Unpaired *t*-test
S-LP 1: 6.46 (1.12), *N*=13	11.88 (2.66), *N*=10	*t* (21) = 6.6465, *p* > 0.0001^*∗*^
S-LP 2: 10.33 (1.30); *N*=9	9.18 (0.78), *N*=9	*t* (16) = 2.2477, *p*=0.03 (NS)
S-LP 3: 4.42 (0.71); *N*=10	5.39 (2.05), *N*=7	*t* (15) = 1.4007, *p*=0.18 (NS)

^*∗*^Significant after Bonferroni correction 0.05/3 = 0.01; NS = not significant.

## Data Availability

The data used to support the findings of this study have not been made available because they are restricted by the Health Sciences Research Ethics Board (University of Toronto) in order to protect patient privacy.
